# Endometrial Infusion with Plasma Rich in Growth Factors (PRGF) in IVF Cycles: Randomized Clinical Trial in Very Thin Endometrium and Observational Uncontrolled Follow-Up After the Randomized Clinical Trial

**DOI:** 10.3390/jcm14061952

**Published:** 2025-03-13

**Authors:** Ines Castells, Marcos Ferrando, María de la Fuente, Maitane Gantxegi, Fernando Quintana, Juan Manuel Mascaros, Eduardo Anitua, Roberto Matorras

**Affiliations:** 1 Instituto Valenciano de Infertilidad (IVI), IVIRMA, 48940 Bilbao, Spain; ines.castells@ivirma.com (I.C.); marcos.ferrando@ivirma.com (M.F.); fernando.quintana@ivirma.com (F.Q.); 2 BTI—Biotechnology Institute ImasD, 01007 Vitoria-Gasteiz, Spain; maria.delafuente@bti-implant.es (M.d.l.F.); eduardo@fundacioneduardoanitua.org (E.A.); 3 University Institute for Regenerative Medicine & Oral Implantology, UIRMI (UPV/EHU—Fundación Eduardo Anitua), 01007 Vitoria-Gasteiz, Spain; 4 Biobizkaia Health Research Institute, 48903 Baracaldo, Spain; maitane.gantxegirodriguez@bio-bizkaia.eus; 5 Fundación IVI, IVIRMA, 46004 Valencia, Spain; 6 Human Reproduction Unit, Cruces University Hospital, 48903 Baracaldo, Spain; 7 Obstetrics and Gynecology Department, University of the Basque Country, 48940 Leioa, Spain

**Keywords:** thin endometrium, endometrial thickness, PRP, PRGF, pregnancy rates, live birth rates

## Abstract

**Objectives**: To assess if the instillation of plasma rich growth factors (PRGFs) improves endometrial thickness (EMT) in frozen embryo cycles performed under hormone treatment where the endometrium was very thin (≤5 mm). **Methods**: First, a randomized controlled trial (RCT) was performed comparing women only receiving an increase in estrogen therapy (n = 9) and women receiving both the increase in estrogen therapy and three instillations of PRGF (n = 13). The second part of the study consisted of a prospective observational follow-up of the patients included in the RCT (for 1–3 months in the study group, and for 1–6 months in the control group). **Results**: In the RCT, there was an increase in EMT in both the PRGF and control groups. However, the increase was significantly higher in the PRGF group (1.30 ± 0.67 mm) compared to the control group (0.58 ± 0.51 mm). In the PRGF group, 23% achieved an EMT of 7 mm compared to 0% in the control group. There were 2 pregnancies in the PRGF group resulting from the 3 transfers performed in that group. No transfer was carried out in the control group. There was a significant increase in EMT 1–3 months after the PRGF cycle. The live birth rate per transfer was 20% in the cycles following the PRGF cycles, whereas it was 30% per starting woman. For women in the control group who later underwent PRGF, the LBR was 57.1%. **Conclusions**: PRGF instillation in cases of very thin endometrium increases EMT moderately. It is suggested that the beneficial effect of PRGF may persist for 1–3 cycles after instillation.

## 1. Introduction

Embryo implantation is one of the most inefficient steps in assisted reproduction [1]. At the time of implantation, both the embryo and the endometrium must be in suitable condition. Vaginal ultrasound (VUS) is the first diagnostic test used to assess the state of the endometrium, and VUS can reveal various conditions that have been related to implantation failure, such as myomas, polyps, synechiae, and malformations, where several therapeutic strategies appear to improve outcomes. Furthermore, VUS is pivotal for assessing endometrial thickness (EMT). Although controversial, a number of studies suggest that a thin endometrium (TE) hinders implantation [2,3,4,5,6] and pregnancy outcomes [7,8,9]. The most commonly used cut-off point to define a TE is an EMT below 7 mm [5].

In some cases of TE, a cause can be found such as adhesions, curettage, and other intrauterine surgeries, infections, radiation, and Müllerian anomalies [10,11,12,13,14]. However, in many cases, no cause is evident [11]. In order to improve pregnancy rates in TE, more than 15 different complementary treatments have been proposed, with controversial benefits [5,15,16]. The incidence of TE in IVF ranges from 1% to 2.5% in most studies [4], while the incidence of EMT < 5 mm ranges from 0.1 to 0.2% [3]. EMT < 5 mm seems to have a more detrimental effect on pregnancy rates than EMT < 7 mm [3,17,18].

In recent years there has been increasing interest in applying the benefits of platelets in medicine. In addition to their involvement in coagulation, platelets contain growth factors and cytokines that can stimulate tissue regeneration or healing in the treated area. Platelet-rich plasma (PRP) is a biological product defined as a portion of the plasma fraction of autologous blood with a platelet concentration above the baseline.

Autologous PRGF (plasma rich in growth factors) technology is characterized by a moderate platelet concentration of 2 or 3 times higher than basal, the absence of leukocytes and inflammatory proteins, and the use of calcium chloride as a platelet activator to initiate the process of platelet degranulation and the liberation of growth factors. PRGF has a balanced content of plasma and platelet growth factors released after this activation. PRGF efficacy has been extensively demonstrated in multiple medical areas such as sports medicine, dentistry, and dermatology [19,20,21,22,23].

Several reports have recently been published on the use of PRP/PRGF in reproductive medicine in the endometrium [20,24,25], ovaries [26,27], and sperm [28]. In-vitro treatment with PRGF increases human endometrial fibroblast proliferation and migration and increases VEGF secretion and endometrial matrix remodeling [20]. Intrauterine administration of activated PRP in a murine model stimulates and accelerates the regeneration of the disturbed endometrium [29]. There are some uncontrolled cohort studies reporting an increase in endometrial thickness after PRP infusion in frozen embryo cycles [30,31,32,33]. This has also been demonstrated in a randomized study [34].

Two recent meta-analyses have shown that PRP was associated with increased pregnancy rates in IVF [24,25]. However regarding PRP and endometrial thickness, in one meta-analysis [24], including two RCT [34,35], an association was found [24], while in the other [25], the meta-analysis was not possible as only one high-quality study was included [35].

The aim of our study was to evaluate the impact of PRGF in EMT by means of an RCT directed at cases with very thin endometrium (VTE), defined as EMT ≤ 5 mm, where pregnancy rates are particularly poor [3,17,18].

## 2. Materials and Methods

Our study included 22 women undergoing frozen embryo transfer (FET) and hormone replacement therapy at our center, in which a very thin endometrium (VTE) was detected over a two-year period. Inclusion criteria were (a) female aged between 18 and 49 years, which corresponds to the age range of the patients treated in our clinic, (b) BMI between 18 and 35, (c) normal standard blood tests (hematocrit, formula, and blood count, general blood biochemistry, infectious serology) in the last 6 months, (d) completion of one FET cycle with own or donor oocytes, (e) use of hormone replacement therapy, (f) EMT ≤ 5 mm despite 10 days of standard doses of estrogen therapy (6 mg/day of estradiol valerate). Exclusion criteria were as follows: (a) history of pelvic inflammatory disease, (b) history of sexually transmitted disease (c) endometrial disease (d) systemic disease that could interfere with PRGF administration (bleeding disorders, thrombocytopenia), and (e) risk of infection.

Table 1 shows the main characteristics of our population. Mean age was 39.14 ± 4.25; 86.4% had had previous failed transfers (27.27% 3 or more previous failed transfers); 45.45% had had clinical miscarriages (9.1% two or more previous miscarriages); and 40.9% had had operative hysteroscopy (HSC). In 54.55% there was a history of at least 1 relevant disease.

This paper presents two different studies, corresponding to the same baseline population: (1) a randomized controlled trial (2) an observational cohort study of the transfers carried out on women from the initial population who did not give birth in the randomized controlled trial (RCT).

### 2.1. Randomized Controlled Trial

**Figure 1:**
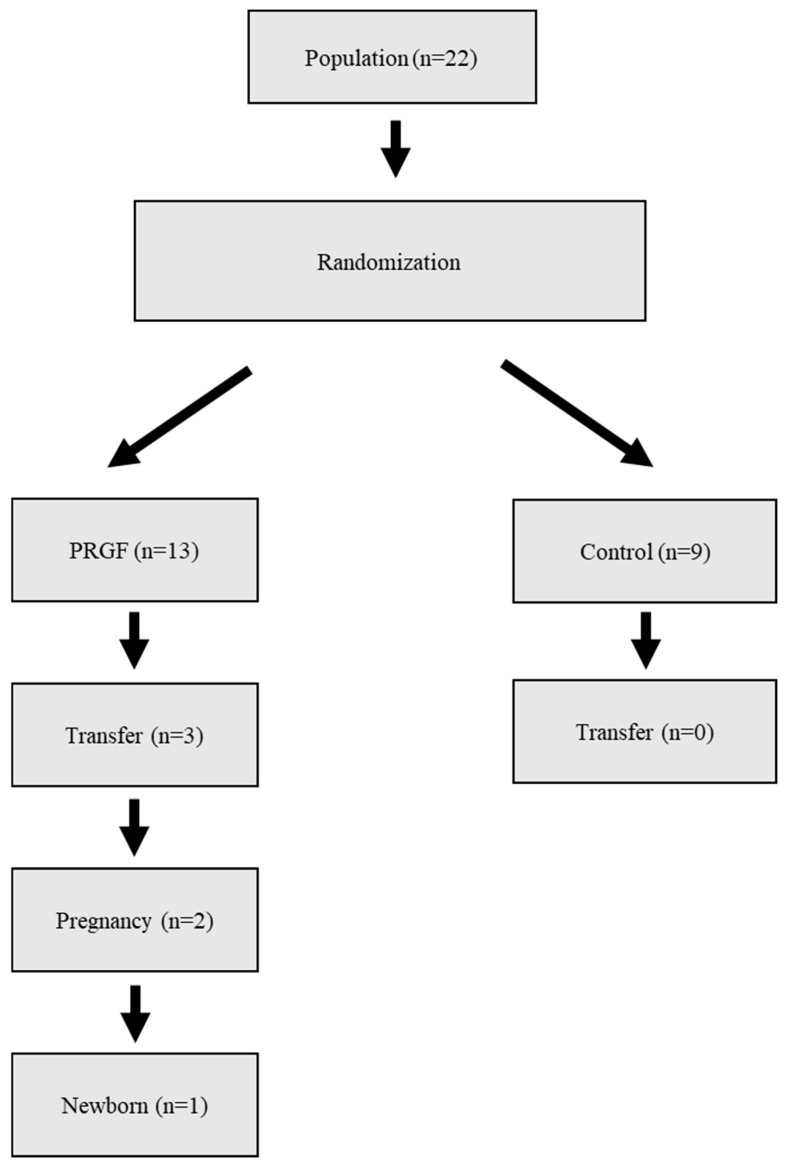
Flow chart of the randomized study.

The study was approved by our Institutional Committee CEIm 2,016,030 and registered in the clinical trial registry under EudraCT code 2016-001716-38. Informed consent was obtained from all women involved in the study. Figure 1 shows the flow chart of the RCT.

**Figure 2:**
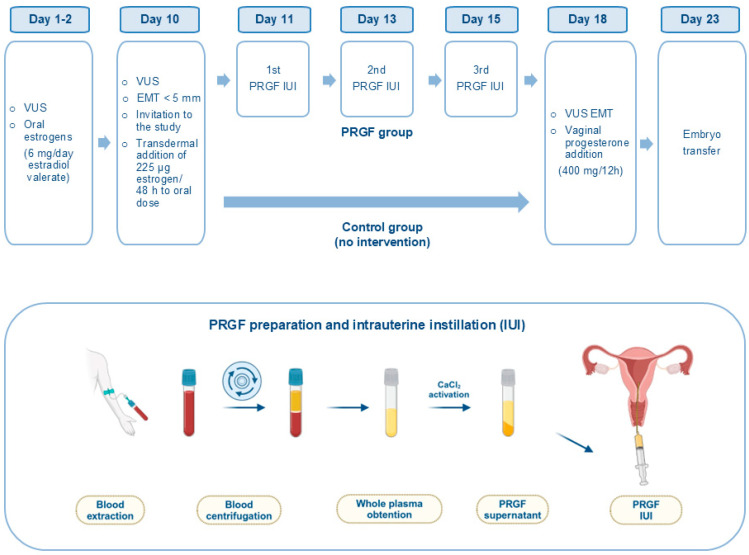
Cycle management in the randomized trial. Diagram of study groups (PRGF and control) with hormone treatment and intervention times. Overview of PRGF preparation and intrauterine instillation. Created by BioRender.com.

All women included in the study were on hormone replacement therapy. Cycle management was as follows: on the 1st–2nd day of the menstrual cycle, a VUS was performed to assess the absence of ovarian follicles > 10 mm. Once ovarian quiescence was verified, endometrial preparation was started with oral estrogens (6 mg/day of estradiol valerate). Ten days after starting estrogen therapy, a VUS was performed (Figure 2).

EMT was assessed in the median longitudinal axis of the uterus as the maximum distance from a basal endometrial interface through the endometrial canal to the opposite endometrial–myometrial interface. EMT measurements were always performed with an empty bladder. EMT was measured with a GE Voluson P6 (General Electric Healthcare) using a 6.5-Mhz vaginal probe (RIC5-9W-RS), and the VUS was performed on each patient by the same gynecologist. If the EMT was ≤5 mm, women were invited to participate in the study, and informed consent was given. The next day, those who accepted were randomized into two groups using a computer-generated random number list; a PRGF group and a control group. In both groups, the dose of estrogen was increased by adding 225 mcg of estrogen/48 h transdermally to the oral dose, and the VUS was repeated 7 days later. In addition, women randomized to receive PRGF underwent PRGF infusion on three occasions (1, 3, and 5 days after invitation to the study). No intrauterine intervention was performed in the control group. On day 7 after the inclusion in the study, depending on the EMT, patients (PRGF and controls) had the following three options: (1) to proceed with embryo transfer if the EMT was ≥7 mm, (2) to proceed with embryo transfer even if the EMT was <7 mm, and (3) to cancel the cycle. In the randomized trial, none of the patients with an endometrial thickness < 7 mm opted for embryo transfer, neither in the PRGF group nor in the control group. Once it was decided to proceed with the embryo transfer, vaginal progesterone (400 mg/12 h) was started and an ultrasound-guided transfer of a single embryo was performed on day 5. An hCG blood test was performed 11 days after ET. If positive, a vaginal ultrasound was done at 5–6 weeks of gestation and again at 7–8 weeks.

PRGF infusion was performed under abdominal ultrasound guidance, using an “embryo transfer catheter” (Frydman^®^ Ultrasoft Echo, Laboratoire CCD, Paris, France). Special care was taken to avoid touching the uterine fundus or injuring the cervix. In cases of excessive vaginal discharge, the vagina was cleansed with saline before aspiration.

To obtain PRGF, 18 mL of venous blood was collected from every woman in sodium citrate tubes. The tubes were centrifuged at 580× *g* for eight minutes at room temperature in a PRGF system centrifuge (BTI Biotechnology Institute, S.L., Álava, Spain) [36]. The whole plasma column was aspirated avoiding the buffy coat containing the leucocytes. After the activation of PRGF with calcium chloride (10% weight/vol) and clot retraction, a volume of 0.5 mL of the PRGF supernatant preparation was infused on each of the three instillation days (Figure 2). The first instillation was done with a fresh sample, while the second and third instillations were performed with thawed PRGF preparations [37,38].

#### Sample Size

Assuming an alpha risk of 0.05 and a beta risk of 0.2 in a two-sided test, and a case/control ratio = 1, 22 subjects were required to detect a difference greater than or equal to 1 mm as statistically significant. The common standard deviation was assumed to be 0.8. A dropout rate of 0% was assumed. Although the live birth rate (LBR) should be the main objective in assisted reproduction studies, this parameter was not used for the predetermination of the sample size because of the large population size that would have been necessary. Indeed, to obtain a 10% difference in the LBR, with an α of 0.05 and a power of 0.8, 586 cycles with very thin endometrium would have been necessary. Since thin endometria are found in 0.1–0.2% of all cycles, the source population would have had to be 297,000–586,000 cycles.

### 2.2. Observational Cohort Study

In this study, we describe the evolution of RCT patients in cycles performed 1 to 6 months after the RCT for the patients in the RCT control group, and 1 to 3 months after the RCT for patients in the PRGF group since no transfers were performed in this group between the 4th and the 6th month. In both cohorts, in cases where the endometrial thickness was <7 mm, the patients were given the option of either carrying out the embryo transfer in spite of this or canceling the cycle

**Figure 3:**
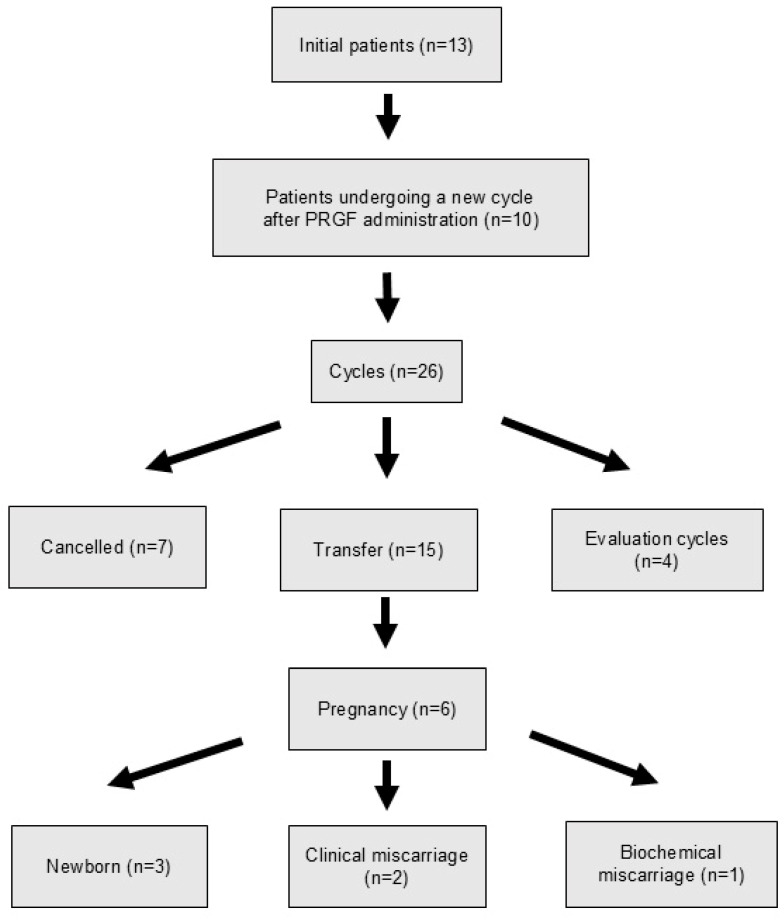
Follow-up of the group receiving PRGF 1 to 3 months after the RCT. The evaluation cycles correspond to cycles with the same hormonal preparation as the transfer cycles but with the objective of performing an endometrial analysis (receptivity, pathology, or microbiology).

The study was approved by our Institutional Review Board under code EOM2022077. Of the 13 women in the randomized PRGF group, 10 women repeated a new cycle (26 cycles initiated, 15 cycles with transfer) (Figure 3).

**Figure 4:**
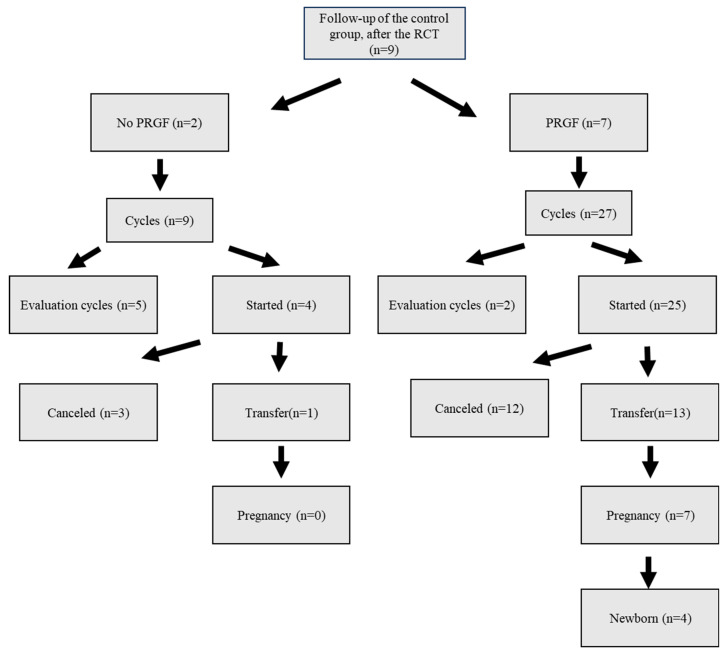
Follow-up of the control group (not receiving PRGF at the RCT) 1 to 6 months after the RCT. The evaluation cycles correspond to cycles with the same hormonal preparation as the transfer cycles but with the objective of performing an endometrial analysis (receptivity, pathology, or microbiology).

Of the 9 women in the control group, all 9 repeated at least one cycle: 7 of them (not randomly) received PRGF (27 cycles initiated, 25 with intention to transfer, and eventually 13 subjected to transfer) and 2 of them did not receive PRGF (9 cycles initiated, 4 of them with intention to transfer, and ultimately only 1 subjected to transfer) (Figure 4).

Of the 66 cycles in the observational study (11 evaluation cycles and 55 cycles with intention to transfer), in 5 cases a natural cycle was used, whereas in the remaining 61 cycles, a new hormone replacement cycle was performed, all starting with a higher dose of estrogen. The route of estrogen administration was changed in 19 cases.

### 2.3. Statistical Analysis

The normality of continuous variables was analyzed using the Kolmogorov–Smirnov test. Comparisons between the two groups were made using the T-test for variables with a normal distribution, otherwise the Mann–Whitney U test was used. Categorical variables were analyzed using the chi-squared test or Fisher’s exact test.

## 3. Results

### 3.1. Demographic Characteristics of Patients

The PRGF and control groups had similar demographic characteristics (Table 1). In the PRGF group, there was one case of each of the following preexisting medical conditions: hyperthyroidism, Hashimoto’s thyroiditis, leukemia (with prior chemotherapy), diabetes, breast cancer (with prior chemotherapy), antiphospholipid syndrome, and microprolactinoma. In the control group, the following conditions were present or had occurred: rectal cancer (with prior chemotherapy and radiotherapy), hyperthyroidism (with prior radioactive iodine treatment), antiphospholipid syndrome, and in two cases, hypothyroidism. The chemotherapy—embryo transfer interval was always longer than 5 years.

In the PRGF group, four women underwent operative hysteroscopy, including one adhesiolysis, one septal excision, one uterine cavity enlargement, and one myomectomy with uterine cavity enlargement. In the control group, there were five operative hysteroscopies, including one curettage, one polypectomy, two metroplasties (T-shaped uterus), and one adhesiolysis.

### 3.2. Evolution of Endometrial Thickness in the RCT

In the control group, a non-significant increase in EMT was observed at randomization (4.29 ± 0.88 mm) and after 7 days of increasing the dose of estrogen therapy (4.87 ± 0.76 mm) (*p* = 0.15). Furthermore, in the PRGF group, EMT increased significantly from 4.44 ± 0.40 mm at randomization to 5.74 ± 0.87 mm, 7 days after receiving the 3 doses of PRGF and the increased dose of estrogen (*p* = 0.001).

There was no difference in EMT between the PRGF and control groups at the time of randomization. However, after 7 days, EMT was significantly increased in the PRGF group (*p* = 0.022). In this respect, the increase in EMT was significantly higher in the study group (1.30 ± 0.67 mm) than in the control group (0.58 ± 0.51 mm), (*p* = 0.01).

None of the nine patients in the control group reached the 7 mm cut-off, compared to 23.08% (3/13) in the PRGF group (*p* = 0.24) (Table 2).

A subanalysis was performed according to the presence or absence of previous surgical HSC. In women with previous operative HSC, mean endometrial thickening was 1.50 ± 0.63 mm in the PRGF group versus 0.173 ± 0.15 mm in the control group (*p* = 0.02). In women without previous HSC surgery, the mean endometrial thickening was 1.22 ± 0.72 mm in the PRGF group versus 0.68 ± 0.54 mm in the control group (*p* = 0.17).

When the subanalysis was restricted to women with or without previous curettages, a similar trend was observed although it did not reach statistical significance. In women with previous uterine curettage, the mean endometrial gain was 1.44 ± 0.8 mm in the PRGF group versus 0.53 ± 0.42 mm in the control group (*p* = 0.12). In women without previous curettage, the mean endometrial increase was 1.23 ± 0.64 mm in the PRGF group and 0.58 ± 0.60 mm in the control group (*p* = 0.08).

### 3.3. Embryo Transfer and Embryo Transfer Outcome in the RCT

None of the patients in the control group underwent embryo transfer; all of them declined due to poor prognosis. In the PRGF group, three transfers were performed, two of which were β-hCG positive. Of these, one resulted in a biochemical miscarriage and the other in a healthy-term newborn (Figure 1).

### 3.4. Follow-Up After the RCT Study

Of the 13 patients randomized to receive PRGF, 10 repeated a new FET cycle, between 1 and 3 months after PRGF instillation. In this group, no embryo transfers were performed between the 4th and 6th month for the following reasons: ongoing pregnancy (n = 4), post-miscarriage recovery (n = 1), additional studies (n = 1), no more embryos available (n = 5), two of which were also included in ongoing pregnancies. Of the 22 FET cycles initiated, 15 embryo transfers were performed (Figure 3). EMT was significantly higher than in the cycle in which PRGF was administered (6.59 ± 1.11 mm versus 5.74 ± 0.87 mm) (Table 3).

There were six pregnancies, three of which resulted in a healthy newborn. The live birth rates were 30% (3/10) per woman, 20% (3/15) per transfer, and 13.6% (3/22) per cycle started (Table 4).

Finally, of the 13 women who received PRGF in the randomized trial, pooling the results of the RCT and of subsequent cycles, the following data were obtained for LBR: 30.8% (4/13) per woman, 22.2% (4/18) per transfer, and 11.4% (4/35) per started cycle (Table 5).

In the control group, all nine women started a new FET cycle. Of these, seven opted to receive PRGF, while two underwent a new FET without PRGF (Figure 4). Both EMT and thickening were significantly higher in women who received PRGF than those who did not (1.60 ± 1.18 mm versus 0.60 ± 1.88 mm, respectively, Table 5). In women who received PRGF secondarily, the following LBR values were obtained: 57.1% (4/7) per woman, 30.8% (4/13) per transfer, and 16.0% (4/25) per cycle started (Table 4). Of the two women in the control group who repeated a cycle without PRGF, only one transfer was performed and no pregnancy was achieved.

### 3.5. Analysis of Side Effects

There were no reports of bleeding, infection, or other complications.

## 4. Discussion

On the maternal side of implantation, TE is a relatively common problem. Although there is no universal agreement regarding the definition of TE, it is generally accepted that TE affects IVF rates [7,8,9]. However, a recent review of the literature concluded that the available evidence does not appear to support a change in management simply because EMT is below an arbitrary threshold [39,40]. Studying the effect of any treatment on TE raises three methodological issues. First, the best way to assess endometrial improvement would be to analyze the pregnancy rates, but such a study would require a very large number of patients to adjust for the embryo factor. Secondly, focusing only on the endometrium might show endometrial changes without affecting IVF outcomes. Finally, the effect would probably vary depending on the threshold used to define TE.

Previous studies on PRP in TE differ in the preparation of PRP, the number of instillations performed, and the days on which the instillations were performed and were directed towards EMT < 7 mm [31,32,33,34,35]. In our protocol, PRGF was chosen over other PRPs because it is a technology with great versatility that allows for various formulations depending on the therapeutic need and controlled activation that facilitates the release of all platelet and plasma factors. In addition, it has a moderate concentration of platelets and avoids inflammatory complications due to the absence of leukocytes and erythrocytes. PRGF was applied in three instillations on days +1, + 3, and +5.

Our investigation was directed to very thin endometrium (VTE) rather than just thin endometrium (TE). VTE has a reported frequency of 0.1–0.2%, compared to 1–2.5% of TE [3,4]. The impact on pregnancy rates of VTE is considerably greater than that of TE [3,4,17,18,41,42]. Moreover, it could be speculated that they might have a different etiopathogenesis, as suggested by the very high rate of associated conditions present in our population (59.1%), with three of the patients previously receiving chemotherapy for cancer and one case receiving radioactive iodine, as well as the high rate of previous operative hysteroscopies (40.9%). Our main objective was to evaluate the impact of PRGF administration on EMT in VTE. We observed a significant increase of 1.30 ± 0.67 mm in the PRGF group compared to 0.58 ± 0.51 mm in the control group, which is consistent with previously published work [31,33,35,38]. Thus, our data suggests that PRGF administration promotes endometrial development, presumably through the action of some of the morphogens it contains, such as platelet-derived growth factor, vascular endothelial growth factor, insulin-like growth factor, transforming growth factor beta (TGF-β), epidermal growth factor, and fibroblast growth factor [20]. Receptors for several of these have been found in the endometrium [43]. However, it should be noted that only 23% of our PRGF cycles reached 7 mm, considering the limit of non-thin endometrium, compared to none in the control group.

Our results contradict previous studies, in which the EMT reached 7 mm in almost all treated patients [31,33,35]. However, it should be stressed that while in most of the aforementioned studies, the inclusion criteria was <7 mm, in our study the inclusion criteria was ≤5 mm. The main reason for choosing the 5 mm cut-off point was the low pregnancy rate in these cases [3,4,17,18,40,42]. Furthermore, the 5 mm cut-off precluded reaching the 7 mm cut-off simply because of intra-observer variability, which has been reported to be 0.6 ± 0.7 mm [43].

There are a few previous uncontrolled studies reporting pregnancy rates close to 100%, all in small populations and with the 7 mm cut-off point as inclusion criteria [31,32,33]. There are two previous randomized controlled studies on TE; both showed a trend towards better pregnancy rates but did not reach statistical significance [34,35]. Meta-analysis results are contradictory; one of them, including the two previously cited papers [34,35], reported a significantly higher pregnancy rate [24], whereas the other [25] could not be meta-analyzed as only one publication was retrieved [35], while the other was considered at high risk of bias [34].

In terms of pregnancy rates, in our RCT, no patients in the control group achieved embryo transfer, and only three in the PRGF group did. Although pregnancy rates in the PRGF group were good, the reduced sample size precluded statistical analysis. One aspect that was analyzed in our investigation was the follow-up in the cycles after PRGF instillation. To our knowledge, this issue has not been analyzed previously, probably because in the previously reported series (with the 7 mm threshold), almost all cases achieved pregnancy. Interestingly, we observed that in the cycles after the PRGF cycle, the EMT was higher than in the PRGF cycle itself. Although we cannot rule out a regression toward the mean phenomenon or, perhaps improvements due to small changes in hormonal protocols, it deserves further study. If this is confirmed, it should be investigated whether the optimal time for embryo transfer would not be during the PRGF instillation cycle itself. Furthermore, it is noteworthy that of the 13 women who received PRGF in the randomized study, 10 performed additional cycles after randomization, with a live birth rate of 30% per woman and 20% per transfer.

It may therefore be speculated that the effects of PRP/PRGF persist for more than one cycle. In cases of thin endometrium, a number of vascular changes have been reported, such as impaired vascular development, decreased vascular endothelial growth factor expression, and elevated uterine blood flow impedance [44]. It could be suggested that the changes in fibroblast proliferation and migration, VEGF expression, and endometrial matrix remodeling that we described in vitro [20] could persist throughout an additional cycle.

Eventually, the proportion of women who achieved a live newborn, considering together women who received PRGF in the RCT (in the RCT and post-RCT cycles) and those who received PRGF after the end of the RCT, was 40%, which although lower than that in our general population, in our opinion is remarkable for women with EMT ≤ 5 mm. Previously reported LBRs in the EMT < 5 mm range from 0 to 25.9% in one series [3] to 15.6% [17] and 18% in another [18].

It is concluded that in VTE, co-administration of PRGF with increased estrogen dosage enhances EMT significantly more than increasing the estrogen dosage alone. However, only a few reached the 7 mm threshold. During follow-up, it was observed that PRGF administration was associated with an increase in EMT compared to EMT in the cycle in which PRGF was administered. It is suggested that PRGF may have a long-lasting effect of more than 1 cycle.

Finally, the pregnancy rates and LBR obtained among women who received PRGF at some point (in the RCT or the observational study) were remarkable, considering the very poor prognosis expected in VTE. Based on the increased EMT, the considerable pregnancy rates, and the absence of adverse effects, in our opinion, PRGF should be considered as an adjuvant therapy in cases of VTE.

### 4.1. Strengths of Our Study

We targeted VTE, where small increases in EMT may have a beneficial effect. We also avoided including patients with EMT close to 7 mm, where the cut-off point could be exceeded by intra-observer variability alone.

Additionally, the fact that our work focused on EMT excluded the influence of a number of confounding variables, especially those related to embryonic factors.

However, embryonic factors play an important role in implantation and should be taken into account in all studies that consider pregnancy rates. Finally, our follow-up study, in addition to the RCT, provides real-world data, with significant pregnancy and live birth rates achieved in such a poor prognosis population.

### 4.2. Limitations of Our Study

The limitations of our work include the fact that in our study, although randomized, VUS for EMT was not blinded. Therefore, it cannot be discarded that the investigator involved gave a somewhat higher EMT to patients receiving PRGF. Consistent with this, in a non-randomized study using PRP, higher EMTs were given when the gynecologist performing the VUS was not blinded than when they were blinded [45].

Furthermore, EMT has a well-known inter-observer and intra-observer variability [43,46], but this problem should be corrected when applying statistical calculations.

The main objective of our study, to assess the increase in EMT, was demonstrated. However, the clinical relevance of an increase in EMT of 1.30 ± 0.67 mm (compared to 0.58 ± 0.51 in the control group) remains to be ascertained. In any case, the fact that ideal EMT was achieved in 30% of VTE patients supports the benefit of PRGF.

The reduced number of patients who underwent embryo transfer (none in the control group) precluded the analysis of pregnancy rates.

In addition to the usual limitations of a non-RCT, the observational study should take into account the possibility of regression to the mean and the hypothetical effect of a change in hormone treatment protocol, although to our knowledge no hormone protocol has been shown to be superior in terms of EMT or pregnancy rates.

## 5. Conclusions

Instillation of PRGF in cases of very thin endometrium results in a moderate increase in endometrial thickness; the beneficial effect of PRGF appears to be prolonged for 1–3 cycles after administration.

### What Does This Study Add to the Clinical Work

The instillation of plasma rich in growth factors into the uterine cavity improves endometrial thickness in cases of very thin endometrium (≤5 mm). More studies are required to assess if the increase in endometrial thickness will significantly increase clinical outcomes.

## Figures and Tables

**Table 1:** Demographic characteristics of the total population, study group, and control group.

	Total (N = 22)	Groups		
		Study (N = 13)	Control (N = 9)	*p*-Value
Age (years)	39.14 ± 4.25 (37.25–41.02)	38.85 ± 4.83 (35.93–41.76)	39.56 ± 3.47 (36.89–42.22)	0.693
Weight (kg)	60.28 ± 6.96 (57.19–63.37)	59.78 ± 8.48 (54.66–64.91)	61.00 ± 4.30 (57.69–64.31)	0.664
Height (m)	1.63 ± 0.07 (1.59–1.66)	1.63 ± 0.08 (1.58–1.68)	1.62 ± 0.07 (1.57–1.67)	0.790
BMI (kg/m^2^)	22.89 ± 3.41 (21.38–24.40)	22.57 ± 3.61 (20.39–24.75)	23.35 ± 3.26 (20.84–25.85)	0.605
Previous transfers				
No transfer	13.64% (3)	23.08% (3)	0.00% (0)	0.121
Own oocyte transfers	50% (11)	53.85% (7)	44.44% (4)	0.308
1	54.55% (6)	71.43% (5)	25.00% (1)	
2	18.18% (2)	14.29% (1)	25.00% (1)	
3 or more	27.27% (3)	14.29% (1)	50.00% (2)	
Donor oocyte transfers	36.36% (8)	23.08% (3)	55.55% (5)	0.915
1	25.0% (2)	33.33% (1)	20.00% (1)	
2	37.5% (3)	33.33% (1)	40.00% (2)	
3 or more	37.5% (3)	33.33% (1)	40.00% (2)	
Previous biochemical miscarriage	13.64% (3)	23.08% (3)	0.00% (0)	0.121
Previous clinical miscarriage	45.45% (10)	46.15% (6)	44.44% (4)	0.937
Previous operative hysteroscopy	18.18% (4)	30.77% (4)	77.78% (7)	0.147
Pre-existing condition	59.09% (13)	53.85% (7)	66.67% (6)	0.548
Smoking habit	4.55% (1)	7.69% (1)	0.00% (0)	0.412

**Table 2:** Endometrial thickness in the study group and the control group.

	Group		
	Study (N = 13)	Control (N = 9)	*p*-Value
Initial endometrial thickness (mm)	4.44 ± 0.40 (4.20–4.68)	4.29 ± 0.88 (3.61–4.97)	0.787
Final endometrial thickness	5.74 ± 0.87 (5.21–6.26)	4.87 ± 0.76 (4.28–5.45)	0.022
Difference in endometrial thickness	1.30 ± 0.67 (0.89–1.71)	0.58 ± 0.51 (0.18–0.97)	0.010
Women with EMT ≥ 7 mm (%)	23.08% (3/13)	0 (0/9)	0.24

**Table 3:** Endometrial thickness follow-up of the patients who were randomized to PRGF 1 to 3 months after the randomization cycle.

		*p*
EMT in cycle after PRGF cycle (mm)	6.59 ± 1.11 (5.94–7.23)	
EMT in PRGF cycle (mm)	5.74 ± 0.87 (5.21–6.26)	
EMT in cycle after PRGF cycle –EMT in PGRF cycle (mm)	0.85 ± 1.07 (0.32–1.56)	0.03
EMT in cycle after PRGF cycle –EMT in PGRF cycle, categorized		
Increase	66.67% (10)	
Decrease	20.00% (3)	
No changes	13.33% (2)	

**Table 4:** Pregnancy rates in the randomized study and the cycles post-randomization.

	PRGF Group at Randomization			Control Group at Randomization		
	PRGF Cycle Randomized Study	Post-Randomization Cycle	Combination	Control Group Randomized Study	Post-Randomization Cycle	
					Receiving PRGF	Not Receiving PRGF
Initial Patients (n)	13	10	13	9	7	2
Per transfer PR (%)	66.7 (2/3)	40 (6/15)	44.4 (8/18)	NC	53.8 (7/13)	0 (0/1)
Per started cycle PR (%)	15.4 (2/13)	22.3 (6/22)	22.9 (8/35)	0 (0/9)	28.0 (7/25)	0 (0/4)
Per starting woman PR (%)	15.4 (2/13)	60 (6/10)	61.5 (8/13)	0 (0/9)	57.1 (4/7)	0 (0/2)
Per transfer LBR (%)	33.3 (1/3)	20 (3/15)	22.2 (4/18)	NC	30.8 (4/13)	0 (0/1)
Per started cycle LBR (%)	7.7 (1/13)	13.6 (3/22)	11.4 (4/35)	0 (0/9)	16.0 (4/25)	0 (0/2)
Per starting woman LBR (%)	7.7 (1/13)	30 (3/10)	30.8 (4/13)	0 (0/9)	57.1 (4/7)	0 (0/2)

**Table 5:** Endometrial thickness follow-up in the control group 1 to 6 months after the randomized study.

	PRGF		
	No (N = 9)	Yes (N = 27)	*p*-Value
Endometrial thickness in cycle after PRGF cycle (mm)	6.05 ± 1.53 (3.62–8.48)	6.53 ± 1.02 (6.10–6.96)	0.579
Endometrial thickness in PRGF cycle (mm)	5.76 ± 0.49 (5.38–6.13)	4.86 ± 0.63 (4.61–5.10)	<0.001
Endometrial thickness procedure—endometrial thickness PGRF (mm)	0.60 ± 1.88 (−2.39–3.59)	1.60 ± 1.18 (1.10–2.10)	0.148
Endometrial thickness procedure—endometrial thickness PGRF categorized			0.005
Increase	22.22% (2)	88.89% (24)	
Decrease	55.56% (5)	7.41% (2)	
No changes	22.22% (2)	7.41% (1)	

## Data Availability

The raw data supporting the conclusions of this article will be made available by the authors on request.
